# Proteomics in neurons and animals: substrates and therapeutic potential of alpha-and beta-secretase

**DOI:** 10.1186/1750-1326-8-S1-O6

**Published:** 2013-09-13

**Authors:** Stefan Lichtenthaler

**Affiliations:** 1 German Center for Neurodegenerative Diseases (DZNE) & Technische Universitaet Muenchen, Germany

## 

The aspartyl protease BACE1 is involved in myelination – through neuregulin-1 cleavage - and is a major drug target for Alzheimer’s disease, where it acts as the beta-secretase cleaving the amyloid precursor protein. However, little is known about other substrates *in vivo.* Recently, we and others identified over 20 new BACE1 substrates in neurons. To elucidate which of them are particularly relevant *in vivo*, a comprehensive proteomic analysis of BACE1-deficient mouse brains and CSF was carried out. A few membrane proteins, including the cell adhesion proteins CHL1 and contactin-2 as well as the APP protein family, were identified as BACE1 substrates *in vivo* and point to a central function of BACE1 in neurite outgrowth and synapse formation. Validation of substrates and detailed characterization of their cleavage was done in primary neurons, CSF and mouse brains. Additionally, the new substrates can be used as biomarkers for the efficacy and potential side-effects of BACE1 inhibitors in clinical trials of Alzheimer’s disease. Mechanistic analysis of substrate cleavage suggests that BACE1 controls the cell surface levels of its substrates and thus their function. Levels of most other proteins were not significantly changed in BACE1-/- brains, suggesting that therapeutic BACE1 inhibition is unlikely to lead to major proteome changes. The talk will also present new substrates and functions for ADAM10 in the brain.

